# Supplementary material for: Association between General Anesthesia and Root Canal Treatment Outcomes in Patients with Mental Disability: A Retrospective Cohort Study
Source: J Pers Med. 2022 Feb 3;12(2):213. doi: 10.3390/jpm12020213 (PMC8876241; doi:10.3390/jpm12020213)
Supplement: Supplementary file 1 [file jpm-12-00213-s001.zip › jpm-1565315-supplementary.pdf]

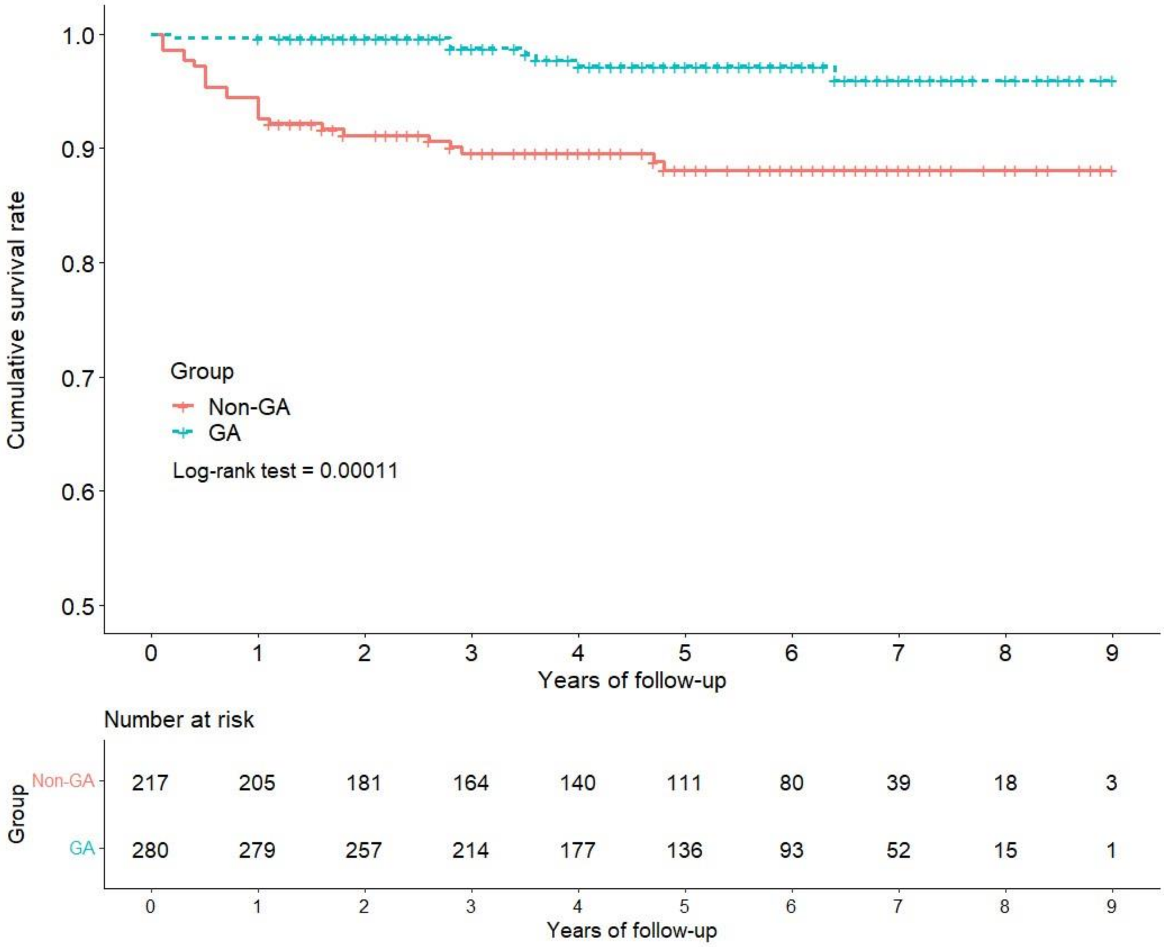

Figure S1. Comparison of 9-year cumulative survival rate of teeth receiving root canal treatment with and without general anesthesia by failure definition of endodontic re-treatment. GA, general anesthesia; non-GA, not general anesthesia.

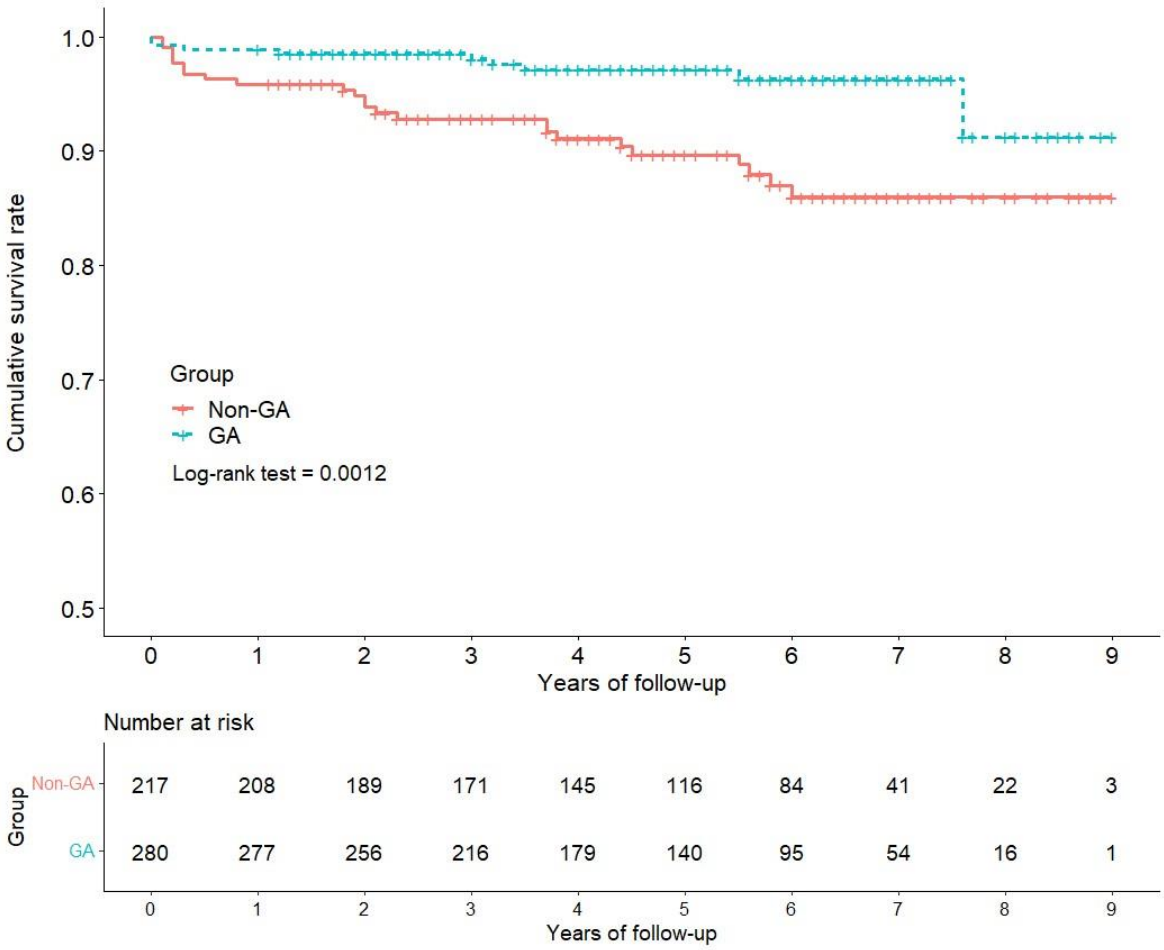

Figure S2. Comparison of 9-year cumulative survival rate of teeth receiving root canal treatment with and without general anesthesia by failure definition of extraction. GA, general anesthesia; non-GA, not general anesthesia.

Table S1. Procedure codes for general anesthesia

general anesthesia	Procedure code
Nitrous oxide-oxygen sedation	96002C
Heavy sedation	96003C
Intravenous or intramuscular anesthesia	96004C
Semi-opened or semi-closed mask inhalation general anesthesia	96017C
Semi-closed or closed-circulative intratracheal intubation general anesthesia	96020C

The code were available at https://www.nhi.gov.tw/query/query2.aspx

Table S2. Procedure codes for root canal treatment and extraction.

Root canal treatment	Procedure code
Canal enlarge and debridement	90015C
One endodontics	90001C
Two endodontics	90002C
Three endodontics	90003C
Four endodontics	90019C
Five endodontics	90020C
**Extraction**	**Procedure code**
Simple extraction	92013C
Complicated extraction	92014C
Odontectomy, simple case	92015C
Odontectomy, complicated case	92016C

The code were available at https://www.nhi.gov.tw/query/query2.aspx

Table S3. ICD-9-CM codes for selected comorbidities

Comorbidities	ICD-9-CM
Periodontitis	523
Hypertension	401-405
Dyslipidemia	272
Diabetes mellitus	250
Ischemia heart disease	411-414

ICD-9-CM, International Classification of Disease, 9th reversion, Clinical Modification

Table S4. Cox regression hazard ratio analysis: univariable and multivariable model of endodontic re-treatment

Variables	No. of teeth with endodontic re-treatment	CHR (95%CI)	*P* value	AHR (95%CI)	*P* value
General anesthesia					
No	24	Reference group		Reference group	
Yes	7	0.22 (0.08-0.58)	0.002**	0.16 (0.06-0.37)	<0.001**
Age		0.94 (0.91-0.98)	0.001**	0.91 (0.87-0.95)	<0.001**
Periodontitis					
No	14	Reference group		Reference group	
Yes	17	1.36 (0.59-3.13)	0.471	1.41 (0.63-3.15)	0.448
Hypertension					
No	31	Reference group		Reference group	
Yes	0	N/A	-	N/A	
Dyslipidemia					
No	28	Reference group		Reference group	
Yes	3	3.80 (1.14-12.73)	0.030*	8.01(1.62-39.60)	0.011*
Diabetes mellitus					
No	28	Reference group		Reference group	
Yes	3	3.76 (1.11-12.74)	0.034*	0.26 (0.06-1.19)	0.082
Disability type					
Mental retard	27	Reference group		Reference group	
Dementia	0	N/A		N/A	-
Autism	3	0.64 (0.19-2.09)	0.457	0.41 (0.13-1.33)	0.139
Chronic mental illness	1	0.26 (0.03-1.92)	0.185	0.60 (0.08-4.59)	0.618

Severity					
Mild	4	Reference group		Reference group	
Moderate	6	0.37 (0.10-1.31)	0.123	0.30 (0.08-1.14)	0.078
Severe	17	0.94 (0.33- 2.71)	0.908	0.79 (0.26-2.44)	0.685
Extreme severe	4	0.39 ()	0.191	0.38 (0.08-1.71)	0.207

CHR=crude hazard ratios; AHR=adjusted hazard ratios; Failure of RCT= event of either endodontic re-treatment or extraction; GA =general anesthesia; N/A= not applicable.

*p<0.05. **p<0.01.

Table S5. Cox regression hazard ratio analysis: univariable and multivariable model of extraction

Variables	No. of teeth with extraction	CHR (95%CI)	*P* value	AHR (95%CI)	*P* value
General anesthesia					
No	24	Reference group		Reference group	
Yes	9	0.30 (0.13-0.70)	0.005**	0.38 (0.16-0.89)	0.025*
Age		1.03 (1.01-1.05)	0.003**	1.01 (0.98-1.05)	0.536
Periodontitis					
No	14	Reference group		Reference group	
Yes	19	1.53 (0.71-3.33)	0.280	1.33 (0.58-3.02)	0.500
Hypertension					
No	32	Reference group		Reference group	
Yes	1	2.65 (0.28-24.82)	0.395	0.89 (0.13-5.99)	0.903
Dyslipidemia					
No	31	Reference group		Reference group	
Yes	2	2.12 (0.36-12.29)	0.403	0.97 (0.27-3.46)	0.958
Diabetes mellitus					
No	32	Reference group		Reference group	
Yes	1	0.99 (0.10-10.03)	0.991	0.56 (0.08-4.20)	0.575
Disability type					
Mental retard	23	Reference group		Reference group	
Dementia	4	4.42 (1.58-12.32)	0.005**	1.77 (0.27-11.47)	0.547
Autism	1	0.24 (0.04-1.57)	0.137	0.26 (0.03-1.95)	0.190
Chronic mental illness	5	1.52 (0.66-5.03)	0.492	0.87 (0.23-3.35)	0.840

Severity					
Mild	2	Reference group		Reference group	
Moderate	17	2.25 (0.46-11.05)	0.317	2.57 (0.48-13.81)	0.273
Severe	10	1.11 (0.22-5.71)	0.900	1.39 (0.25-7.78)	0.710
Extreme severe	4	0.85 (0.15-4.79)	0.849	1.06 (0.18-6.42)	0.947

CHR=crude hazard ratios; AHR=adjusted hazard ratios; Failure of RCT= event of either endodontic re-treatment or extraction; GA =general anesthesia; N/A= not applicable.

*p<0.05. **p<0.01.
